# A case of lymphoma mimicking infected internal iliac artery aneurysm

**DOI:** 10.1186/s40792-023-01665-0

**Published:** 2023-05-18

**Authors:** Yohei Ichikawa, Shinsuke Kikuchi, Hiroya Moriyama, Takamitsu Tatsukawa, Seima Ohira, Yuki Kamikokura, Yuri Yoshida, Mayumi Hatayama, Sayaka Yuzawa, Naoki Wada, Daiki Uchida, Atsuhiro Koya, Nobuyoshi Azuma

**Affiliations:** 1 Department of Vascular Surgery, Asahikawa Medical University, Midorigaoka-Higashi 2-1-1-1, Asahikawa, 078-8510 Japan; 2 Department of Cardiovascular Surgery, Kyorin University Hospital, Tokyo, 181-8611 Japan; 3 Department of Diagnostic Pathology, Asahikawa Medical University Hospital, Asahikawa, 078-8510 Japan; 4 Division of Gastroenterology and Hematology/Oncology, Department of Medicine, Asahikawa Medical University, Asahikawa, 078-8510 Japan; 5 Department of Renal and Urologic Surgery, Asahikawa Medical University, Asahikawa, 078-8510 Japan

**Keywords:** Infected aneurysm, Ruptured aneurysm, Lymphoma, Iliopsoas abscess

## Abstract

**Background:**

Malignant lymphoma rarely mimics an infected arterial aneurysm and a ruptured arterial aneurysm because of similar imaging findings, leading to misdiagnosis. The hematomas of ruptured aneurysms are radiologically difficult to distinguish from those of malignant lymphoma in emergency settings. Hence, a definitive diagnosis is crucial to avoid unnecessary surgery.

**Case presentation:**

A man in his 80s with hematuria and shock vital had right internal iliac artery aneurysm (IIAA) and perianeurysmal fluid retention, which appeared to be a ruptured or an infected aneurysm. Treatment was initiated for infected IIAA instead of for ruptured IIAA. Systemic inflammatory response syndrome developed, and the infectious sources were assessed. Pacemaker lead and urinary tract infections were identified and treated; however, blood pressure was unstable. The aneurysm was treated with endovascular aortic aneurysm repair following antibiotic therapy; however, fluid retention increased, and inflammatory status and hematuria deteriorated. Open surgical conversion was performed to manage the infected lesions. Although an iliopsoas abscess was detected during surgery and nephrectomy and ureterectomy were performed to control the hematuria, analysis of the removed tissues led to the pathological diagnosis of diffuse large B-cell lymphoma (DLBCL).

**Conclusions:**

We encountered a case of DLBCL with imaging findings mimicking an infected internal iliac artery aneurysm, and definitive diagnosis was made more than 2 months after the initial examination. Definitively diagnosing malignant lymphoma around an iliac artery aneurysm based merely on symptoms and imaging findings is extremely difficult. Thus, histological examination should be actively performed in atypical infected aneurysms.

## Introduction

The iliopsoas muscle, ureters, and para-aortic lymph nodes are in proximity to the abdominal aorta, a retroperitoneal organ. Thus, an iliopsoas abscess and retroperitoneal malignant lymphoma need to sometimes be distinguished from an infected abdominal aortic aneurysm (AAA) and hematoma of a ruptured aneurysm [1, 2]. With the aging population, an iliopsoas abscess is no longer a rare condition. It can be differentiated from infected AAAs based on a characteristic imaging finding of abscesses forming within the iliopsoas muscle. Conversely, retroperitoneal malignant lymphomas exhibit no distinctive imaging features; the imaging results of these lymphomas are similar to those of infected aneurysms and contained aortic rupture, particularly when the lesion is close to an aneurysm. Thus, unless a physician has a suspicion and performs histological examinations, many disorders are difficult to distinguish from one another.

Herein, we reported the case of a patient with diffuse large B-cell lymphoma (DLBCL) who was referred to us with a suspected infected internal iliac artery aneurysm (IIAA) and required two laparotomies before a definitive diagnosis could be reached, following which the patient’s life could not be saved.

## Case

**Fig. 1:**
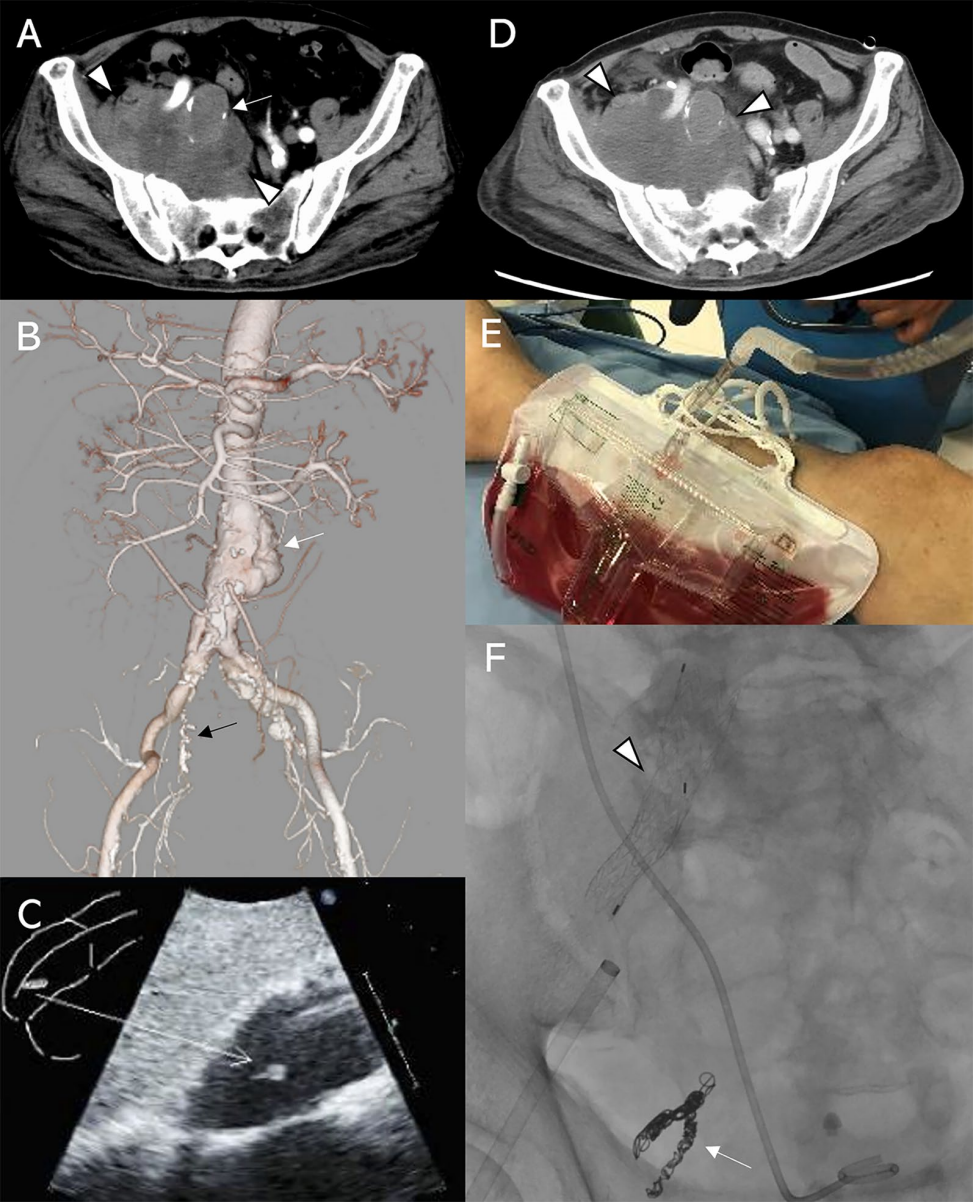
**A** Preoperative computed tomography (CT) revealed a 28-mm right internal iliac artery aneurysm (IIAA, arrow) and perianeurysmal fluid retention (arrowheads). **B** Three-dimensional CT angiography showed abdominal aortic aneurysm (white arrow) and occluded IIA (black arrow). **C** Mobile 15-mm mass was observed at the tip of the pacemaker lead as a source of infection. **D** CT revealed increased perianeurysmal fluid retention on day 18 of hospitalization (arrowheads). **E** Aggravated hematuria with increased perianeurysmal fluid retention. **F** Endovascular aortic aneurysm repair (arrowhead) and coil embolization (arrow)

A man in his 80 s, with hematuria and edema of the right lower extremity had been hospitalized 2 months earlier and received medications for right sciatica. However, the symptoms exacerbated over time, and computed tomography (CT) revealed a 28-mm right IIAA and perianeurysmal fluid retention causing right pyelectasis. He was transferred to our hospital, on an emergency basis with a suspected ruptured IIAA. AAA was also detected, and although the IIAA appeared to have ruptured, the IIA was occluded (Fig. 1A, B). His medical history included pacemaker placement for sick sinus syndrome 22 years ago, percutaneous coronary intervention for angina pectoris 4 years ago, and aortic arch replacement for thoracic aortic aneurysm 1 year ago. At the time of hospitalization, his body temperature, blood pressure, and heart rate were 35.4 °C, 86/58 mmHg, and 61 beats/min, respectively. He showed gross hematuria and prominent edema of the right lower extremity. Blood tests revealed severe inflammation (white blood cell [WBC] count, 12,340/μL; C-reactive protein [CRP], 21.0 mg/dL), anemia (hemoglobin, of 9.9 g/dL), poor nutrition (serum albumin, 1.8 g/dL), and chronic kidney disease (blood urea nitrogen, 41.8 mg/dL; serum creatinine, 1.97 mg/dL). Systemic inflammatory response syndrome developed, and the infectious sources were assessed. Urinalysis revealed turbidity, positive occult blood, and increased WBC count. Through blood culture, *Staphylococcus hominis* was detected in one of four sets. Urine culture revealed the presence of methicillin-resistant *Staphylococcus aureus* and *Enterococcus faecalis*. Echocardiography demonstrated a 15-mm mass in the right atrium with movement at the pacemaker lead (Fig. 1C). The patient was diagnosed with a suspected secondary infected right IIAA related to urinary tract or pacemaker lead infection based on the above test results. Because the patient was strongly suspected of septic shock and not ruptured IIAA, systemic infection control to some extent was planned before radical treatment of the suspected infected aneurysm.

Ureterography revealed no evidence of communication between iliac arteries and right ureter and a ureteral stent was placed on the day of admission. Furthermore, the intravenous antibiotic administration of meropenem and vancomycin was initiated for treating urinary tract infection and pacemaker lead infection. On day 2 of hospitalization, the pacemaker was removed. On day 3, improvements in the WBC count (7030/μL) and CRP level (9.97 mg/dL) were observed following the above interventions. Therefore, systemic antibiotic dosing was planned for approximately 3 weeks before aneurysm treatment. However, low blood pressure was noted on day 18. CT revealed an increase in retroperitoneal fluid retention around the right IIAA, raising a suspicion of ruptured aneurysm and uretero-iliac artery fistula (Fig. 1D). Anemia and aggravated hematuria were also detected (Fig. 1E). Therefore, emergency coil embolization of the right IIA and endovascular aneurysm repair (EVAR) from the right common iliac artery to the external iliac artery (GORE®EXCLUDER®, Gore W L & Associates Inc., DE, USA) were performed to control bleeding and as diagnostic treatment on the same day (Fig. 1F). CT angiography and digital subtraction angiography showed no evidence of contrast enhancement of right ureter and urinary bladder; however, delayed retrograde arterial flow from distal branches of IIA might be associated with increase in fluid retention. Ureterography was not performed as the ureteral stent was already placed.

**Fig. 2:**
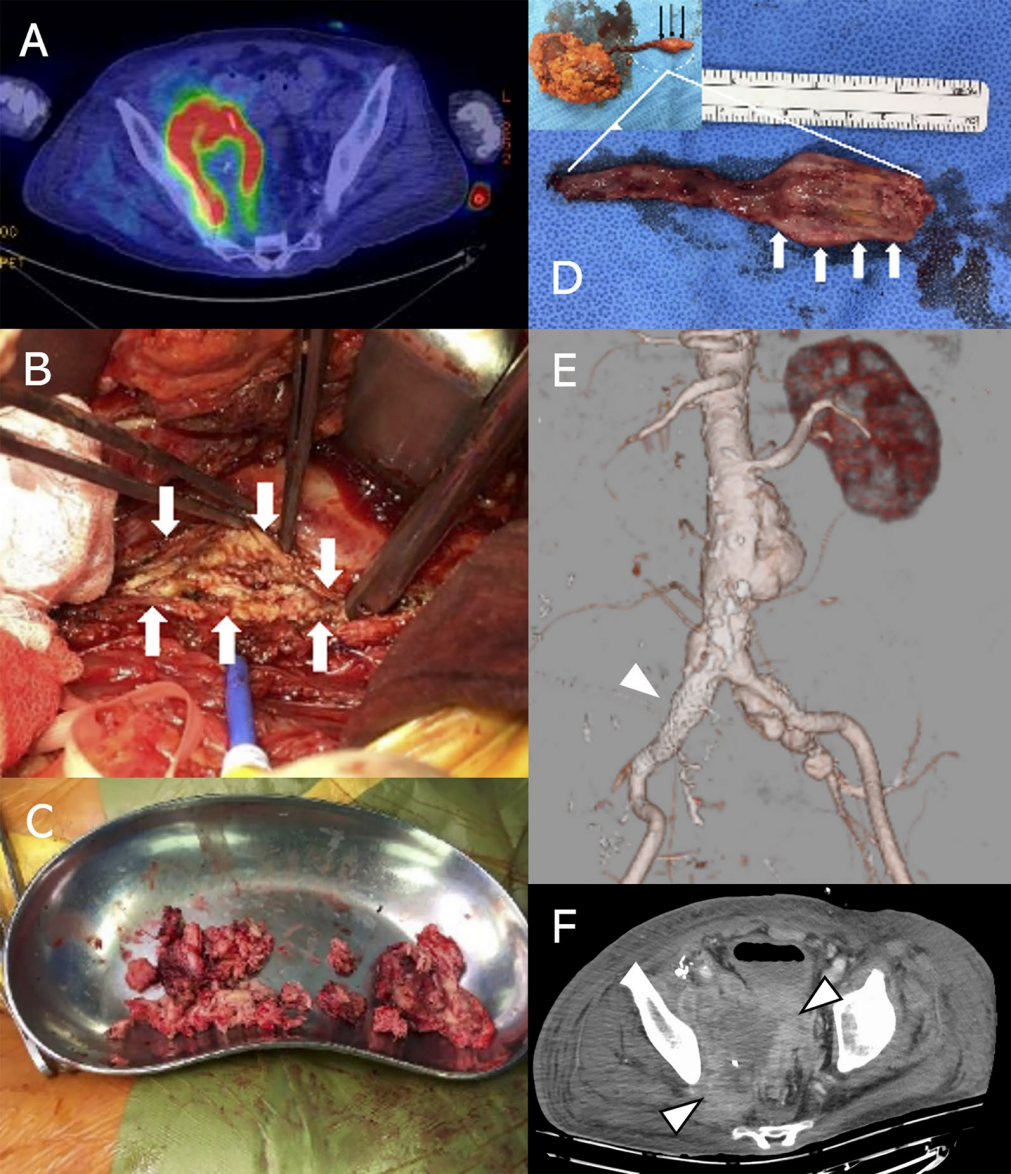
**A** Positron emission tomography–computed tomography (CT) revealed accumulation consistent with retroperitoneal fluid retention around the aneurysm. **B** Intraoperative image of the first laparotomy. The iliopsoas muscle (arrows) had a white cottage cheese-like secretion inside. **C**, **D** Intraoperative image of the second laparotomy. The white necrotic tissues were resected to control infection. The right kidney and ureter were also resected. The distal part of the ureter was enlarged (thin black arrows). No purulent discharge was observed in the lumen, and only wall thickening (thick white arrows) was noted. **E** Postoperative CT angiography after the second laparotomy revealed complete isolation of blood flow in the IIA by endovascular aortic aneurysm repair (EVAR, arrowhead). **F** Pelvic mass grew despite EVAR (arrowheads)

Subsequently, 2-[fluorine-18]-fluoro-2-deoxy-D-glucose (FDG) positron emission tomography (PET)–CT performed on day 21 revealed the accumulation of FDG, consistent with retroperitoneal fluid retention around the aneurysm (Fig. 2A). Based on his medical history and FDG PET–CT data, the patient was diagnosed with an infected aneurysm. EVAR followed by surgical abscess drainage and aneurysmectomy/vascular graft replacement at 1-week interval were scheduled as a two-staged treatment. The proximal part of the abdominal aorta was encircled with Teflon tape following median laparotomy, and the retroperitoneum around the right IIA was incised. No apparent perianeurysmal abscesses were found; however, a mass lesion in the iliopsoas muscle was detected. After this mass was incised, a cottage cheese-like white discharge was identified (Fig. 2B). The contents had serum components and bacterial culture of the components was negative. Intraoperatively, an iliopsoas abscess was clinically diagnosed, and only laparotomy irrigation drainage was performed with drainage tube replacement. However, the patient’s blood test results following the surgery revealed a strong inflammatory response and poor overall condition. Hence, on day 55, gallium scintigraphy was performed to assess residual abscesses, which revealed gallium-67 accumulation along the abscess cavity and ureters. Because the retroperitoneal infection was suspected to have spread to the ureter, the abscesses, ureter, and surrounding tissues were completely debrided (Fig. 2C). Abscess drainage and right nephroureterectomy were performed as the second laparotomy in collaboration with the urology department on day 79. On macroscopic examination, the distal third portion of the ureter was found to be enlarged. An incision was made to the enlarged ureter after nephroureterectomy; no purulent discharge was observed in the lumen, and only wall thickening was noted (Fig. 2D). Following the complete irrigation of the abdominal cavity and extensive debridement of tissues around the kidney and ureter, periarterial tissues were visually examined for infection, and the abdomen was closed as usual. After the second surgery, the pelvic lesion grew despite EVAR and coil embolization having completely controlled blood flow in the left IIA (Fig. 2E, F).

**Fig. 3:**
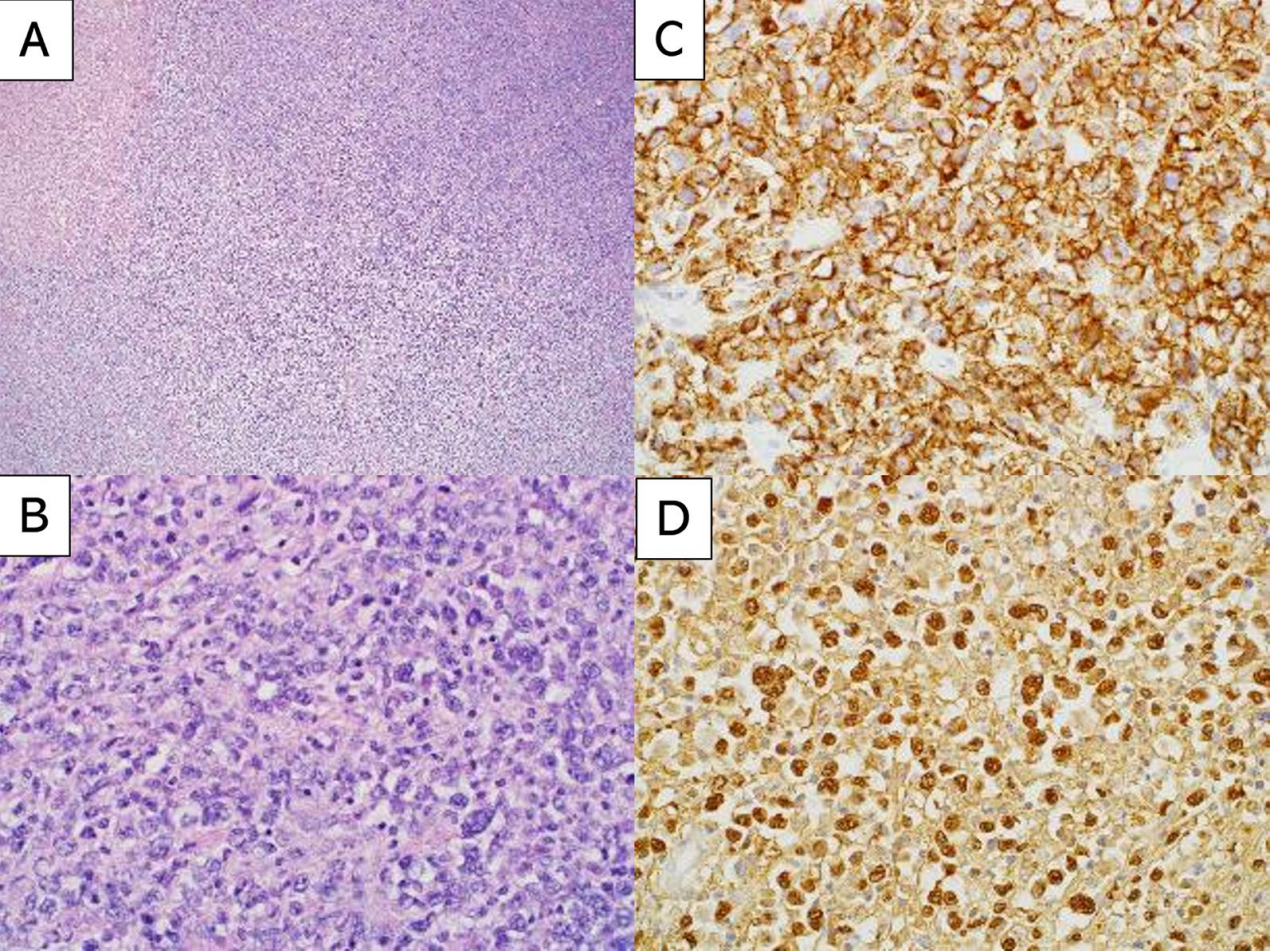
Hematoxylin and eosin (HE) staining, immunohistochemical staining, and Epstein–Barr virus-encoded small RNA (EBER) in situ hybridization (ISH) in the resected perianeurysmal tissues. **A** HE staining (4 ×) showed diffuse growth of atypical cells with necrosis (upper left). **B** HE staining (40 ×) showed medium-to-large atypical lymphoid cells infiltrated in the tissue, suggesting diffuse large B-cell lymphoma. **C** Atypical lymphoid cells were positive for CD20, the classic marker of B-cells (40 ×). **D** EBER–ISH showed positivity in the neoplastic cells (40 ×)

The removed tissues were examined pathologically, which led to the definitive diagnosis of DLBCL (Fig. 3). Following consultation with the hematology department, we agreed that the patient’s poor overall state would make chemotherapy intolerable. The patient’s condition failed to improve, and he died on day 95 of hospitalization owing to multiple organ failure.

## Discussion

The number of patients diagnosed with retroperitoneal tumors has been increasing over time, and malignant tumors are larger and more frequently found in older people [3]. Malignant lymphomas are relatively rare in retroperitoneal tumors, accounting for only 3.5–21.5% of all retroperitoneal tumors [4]. Infected AAAs are also relatively rare, accounting for merely 1.5% of all aortic aneurysms [5]. Therefore, non-Hodgkin’s lymphoma occurring around an AAA or an IIAA is a rare pathological condition. The differential diagnosis between symptomatic aneurysm, such as ruptured aneurysm and non-Hodgkin’s lymphoma, based merely on imaging studies is extremely challenging, especially when the aneurysm and tumor are in close proximity [6, 7]. Because of the similarity in clinical and imaging findings, distinguishing between these two pathological conditions is difficult. Abdominal or lower back pain is associated with mass lesions around the abdominal aorta that extend to the retroperitoneum as well as aneurysm; moreover, distinguishing their clinical symptoms based on the site of pain is challenging. Both non-Hodgkin’s lymphoma and infected aneurysms can be associated with fever, making it difficult to distinguish them based on accompanying symptoms of fever. In the current case, the patient had preceding infectious conditions including urinary tract and pacemaker lead infections. The patient more likely had infective IIAA upon initial examination, and the surgical treatment plan was developed without considering lymphoma.

FDG PET–CT and gallium scintigraphy reveal positive accumulation in non-Hodgkin’s lymphomas [4]; however, the findings are similar to those observed in inflammatory tissues, such as infected aneurysms [8]. Therefore, clearly differentiating non-Hodgkin’s lymphomas from infected aneurysms merely based on imaging results is difficult. Based on these diagnostic difficulties, histological investigations are required to differentiate infective aneurysm from malignant tissues around the aneurysm. In the present case, definitive diagnosis was delayed, because histological investigation had not been performed in the second laparotomy. Tissue culture was only examined at the first laparotomy. DLBCL was unexpectedly diagnosed based on histological findings of the enlarged ureter. In this regard, we might have diagnosed the pathology before surgery. Endosonography-guided fine-needle aspiration is a relatively minimally invasive and simple technique for the histological diagnosis of lymphoma, with a good diagnostic performance and an accuracy of 96–98% [9].

In our practice, it is worth exploring when a definitive diagnosis is difficult to make, because a definitive diagnosis could avoid unnecessary surgery. Valentine et al. reported the clinical results of 16 patients who had nonvascular emergencies presenting as ruptured AAAs [10]. Misdiagnoses in patients who are suspected of having ruptured AAA based on physical findings are relatively uncommon. They mentioned the importance of correcting the primary disease in most misdiagnosed cases [10]. They confirmed definitive diagnosis through laparotomy; 63% of the patients (10 of 16) had intact aortic aneurysms during surgery, and 50% of the patients died in the perioperative period because of primary disease and laparotomy invasiveness. Thus, diagnosing the primary disease using a minimally invasive method is essential. However, hypotension and hematuria appear as a ruptured infected aneurysm, and intervention against IIAA would be unavoidable in such cases. In the present case, the patient's condition was misdiagnosed as iliopsoas abscess; however, chemotherapy would have been difficult to administer even if he had been diagnosed at an early stage, because he was unable to walk independently and had poor overall condition. This was a highly suggestive case that reaffirms the importance of histological examination in treating aneurysms with periarterial lesions. On hindsight, the patient’s symptoms hinted the clinical cause. The patient was admitted and received treatment for sciatica as the chief complaint. At 28 mm, IIAA was not sufficiently large to compress the surrounding tissues, including those of the nerve and ureter, signifying that the disease pathology was not associated with aneurysm-related events, such as the rupture of aneurysm and uretero-arterial fistula. In case who overlapped multiple diseases, lack of differential diagnosis happened and it was important to assess every clinical course, including chief compliant and list of differential diagnoses, and not only focus on the diseases under physician’s specialization.

## Conclusion

We encountered a case of DLBCL with imaging findings similar to those of an infected IIAA, and a definitive diagnosis was made more than 2 months after the initial examination. Making a definitive diagnosis of malignant lymphoma that has developed around an IIAA based merely on symptoms and imaging findings is extremely challenging. Thus, histological examination should be actively performed in atypical infected aneurysms.

## Data Availability

The data used for this case report are available from the corresponding author upon request.

## Abbreviations

IIAA: Internal iliac artery aneurysm
DLBCL: Diffuse large B-cell lymphoma
AAA: Abdominal aortic aneurysm
CT: Computed tomography
WBC: White blood cell
CRP: C-reactive protein
EVAR: Endovascular aneurysm repair
FDG: 2-[Fluorine-18]-fluoro-2-deoxy-D-glucose
PET: Positron emission tomography
